# Sexually transmitted infection (STI) knowledge and perceptions among people in HIV-sero-different partnerships in rural southwestern Uganda

**DOI:** 10.1371/journal.pgph.0002817

**Published:** 2024-01-30

**Authors:** Pooja Chitneni, Moran Owembabazi, Eunice Kanini, Simon Mwima, Mwebesa Bosco Bwana, Christina Psaros, Winnie R. Muyindike, Jessica E. Haberer, Lynn T. Matthews

**Affiliations:** 1 Division of General Internal Medicine and Global Health Equity, Brigham and Women’s Hospital, Boston, Massachusetts, United States of America; 2 Harvard Medical School, Boston, Massachusetts, United States of America; 3 Mbarara Regional Referral Hospital and Mbarara University of Science and Technology, Mbarara, Uganda; 4 School of Social Work, University of Illinois Urbana-Champaign, Urbana, Illinois, United States of America; 5 AIDS Control Program, Ministry of Health, Kampala, Uganda; 6 Department of Psychiatry, Behavioral Medicine Program, Massachusetts General Hospital, Boston, Massachusetts, United States of America; 7 Center for Global Health, Massachusetts General Hospital, Boston, Massachusetts, United States of America; 8 Division of Infectious Diseases, Heersink School of Medicine, University of Alabama at Birmingham, Birmingham, Alabama, United States of America; NYU Grossman School of Medicine: New York University School of Medicine, UNITED STATES

## Abstract

Globally, over one million people acquire curable sexually transmitted infections (STI) each day. Understanding how people think about STIs is key to building culturally appropriate STI prevention and treatment programs. We explored STI knowledge and perceptions in rural, southwestern Uganda to inform future interventions. From August 2020 to December 2020, we conducted individual in-depth interviews among adult men and women (≥18 years) with recent or current personal or partner pregnancy, a history of an STI diagnosis and treatment, and membership in an HIV-sero-different relationship. Interviews explored STI knowledge, perceptions, and barriers and facilitators to engaging in STI care. We used inductive and deductive approaches to generate a codebook guided by the healthcare literacy skills framework in a thematic analysis. Ten men with STI, five of their female partners, eighteen women with STI, and four of their male partners participated in individual in-depth interviews. The median age was 41 (range 27–50) for men and 29 (range 22–40) for women. Sixteen (43%) participants were with HIV. Significant themes include: 1) Participants obtained STI knowledge and information from the community (friends, family members, acquaintances) and medical professionals; 2) While participants knew STIs were transmitted sexually, they also believed transmission occurred via non-sexual mechanisms. 3) Participants associated different connotations and amounts of stigma with each STI, for example, participants reported that syphilis was passed down “genetically” from parent to child. 4) Participants reported uncertainty about whether STIs affected pregnancy outcomes and whether antenatal STI treatment was safe. The complicated nature of STIs has led to understandable confusion in settings without formal sexual healthcare education. Robust counseling and education prior to sexual debut will help allow men and women to understand the signs, symptoms, and treatments necessary for STI cure and to navigate often complicated and overburdened healthcare systems.

## Introduction

Globally, over one million people acquire a curable sexually transmitted infection (STI) each day, with the sub-Saharan African region having the highest age-standardized STI incidence and age-standardized STI disability-adjusted life-years [1, 2]. A recent cross-sectional cohort study in southern Uganda found an STI prevalence of 26% among various populations including those with and without HIV and those living in inland and fishing populations [3]. This high STI prevalence is particularly concerning given that Uganda has one of the highest fertility rates in the world, and STIs cause the greatest morbidity and mortality when transmitted vertically to neonates [4–6]. In prior work in rural southwestern Uganda, we described an STI prevalence of 24% and a six-month STI incidence of 29.0 per 100 person-years among women without HIV considering pre-exposure prophylaxis (PrEP) with pregnancy plans and an STI prevalence of 22% among men with HIV with partner pregnancy plans [7–9].

In recent years, there has been renewed interest in combatting STIs. For example, the World Health Organization (WHO) has made the prevention of mother-to-child transmission of syphilis, HIV, and hepatitis B a top priority [10]. However, STI incidence remains high. This is especially true among pregnant populations, with a recent meta-analysis describing pregnant women in sub-Saharan Africa having a higher incidence of chlamydia and trichomoniasis compared to non-pregnant women [11]. Limited point-of-care (POC) diagnostics in resource-limited settings (RLS) and syndromic management likely contribute to missed diagnoses and ongoing transmission. Other factors, however, are likely at play given the increasing STI incidence in other settings with frequent use of STI laboratory diagnostics, like the US [12]. Knowing the factors leading to STI transmission and gaps in prevention and treatment are crucial to curbing STI epidemics, particularly among populations considering pregnancy.

We conducted qualitative interviews with men and women with previous STIs and recent or planned (partner) pregnancy and a subset of their partners to explore their STI knowledge and perceptions. Topics included sources of information, ideas about transmission, treatment, cure, and the influence of pregnancy on STI treatment-seeking behavior. Understanding how people living with HIV and their partners think about these foundational concepts is key to building culturally appropriate STI prevention and treatment programs, especially in antenatal care centers. Though some of these concepts may be unique to our research site, many of the knowledge gaps and misconceptions are likely prevalent elsewhere.

## Methods

### Study setting and participants

Study methods are reported following the COREQ guidelines [13]. We recruited participants using purposive sampling from the Mbarara Regional Referral Hospital’s (MRRH) Healthy Families Clinical Program in southwestern, rural Uganda [14] from August 2020 to January 2021 where our team works. This program provides HIV sero-different couples counseling and care regarding HIV prevention and safer conception inclusive of etiologic, laboratory STI testing for patients participating in research studies [7–9]. A research assistant screened and recruited participants attending the Healthy Families Clinical Program in person. We also screened and contacted participants via telephone from the Healthy Families Clinical Program who agreed to be contacted for future studies. We planned to recruit up to 40 participants (or until data saturation was reached), stratified by sex and inclusive of both index participants (those with a history of STI) and partner participants [15]. Partner participants were contacted for study inclusion with permission from index participants. The authors did not have access to additional participant-identifying information during or after the study.

Inclusion criteria consisted of men and women age 18 or older with 1) prior, self-reported, or clinically documented, lifetime syndromic or laboratory-diagnosed STI; 2) engaged in the MRRH Healthy Families Clinical Program; 3) recent personal or partner pregnancy within the past three years or pregnancy intentions; and 4) fluency in English or Runyankole (the dominant local language). Exclusion criteria consisted of an inability to provide informed consent.

### Data collection

We developed semi-structured individual, in-depth interview guides (sample questions outlined in **Table 1**) with the aim of exploring STI knowledge, perceptions, and barriers and facilitators to engaging in STI care. Questions were informed by local clinical experience and observation and literature reviews on STI knowledge and care in Uganda and sub-Saharan Africa [7–9, 16–18]. A brief interviewer-administered questionnaire captured socio-demographic information and sexual behavior.

**Table 1:** Sample domains, questions, and probes from individual in-depth semi-structured qualitative interviews.

Domain	Sample questions and probes
Sexually Transmitted Infection (STI) knowledge	Can you tell me what the term STI means to you? ◾ Can you list and/or describe some STIs? ◾ How/Where did you learn about STIs? ◾ Who could you go to with questions regarding STIs?
	What are some of the ways a person can get an STI?
	What methods could a person use to protect themselves from STIs?
	What symptoms are associated with the STIs you listed/described?
Personal history of STI	Have you ever been diagnosed with an STI? If yes, please tell me about this experience. ◾ Which STI were you diagnosed with? ◾ In what setting were you diagnosed?
	How did you feel and react to your previous STI diagnosis? ◾ How did you process or cope with the STI diagnosis? ◾ Tell me about how you were able to talk about this diagnosis and with whom you talked?
	How did a prior STI diagnosis affect or change your sexual behavior?
STI and pregnancy	What are your thoughts about STIs during pregnancy? ◾ How does being pregnant affect the way that you think about having an STI?
	How would you feel if you or your partner had an STI during pregnancy?
	What is your opinion about taking STI treatment during pregnancy?

All interviews were conducted in Runyankole, audio-recorded, and then translated and transcribed into English by a female, college-educated, research assistant, trained in qualitative methods, and fluent in both Runyankole and English. Interviews lasted between 30 to 90 minutes and were conducted in a private research room on the Mbarara University of Science and Technology campus.

### Analysis

We used inductive and deductive approaches guided by the healthcare literacy skills framework in a thematic analysis of the interviews [19]. Four team members reviewed the transcripts to iteratively develop a codebook consisting of emerging categories and definitions. Two team members then double-coded ~20% of transcripts, meeting to refine the codebook and clarify any thematic discrepancies with the goal of achieving validity and reliability. One team member then coded the remaining transcripts with continued input and oversight from the study team. Thematic saturation was achieved once no additional codes or themes emerged from the data and emerged after 37 interviews [20]. Qualitative data were managed and analyzed with the aid of NVivo 1.6.2 software. Quantitative data were analyzed descriptively using STATA 15.1 software.

### Ethics

Study procedures were approved by the Mbarara University of Science and Technology Research Ethics Committee (11/08-19), the Uganda National Council of Science and Technology, and the Mass General Brigham (Massachusetts General Hospital and Brigham and Women’s Hospital) Institutional Review Board (Protocol 2020P001077, Boston, MA). Healthcare facility support was obtained, and participants provided written, voluntary informed consent.

### Inclusivity in global research

Additional information regarding the ethical, cultural, and scientific considerations specific to inclusivity in global research is included in the S1 Text.

## Results

### Participant characteristics

We conducted 37 individual in-depth semi-structured qualitative interviews including 18 female participants, 4 of their male partners, 10 male participants, and 5 of their female partners (**Table 2**). The median age of participants was 33. Sixteen (43%) participants were living with HIV. One-third (34%) of participants reported no current or recent pregnancy in the past three years, 2 (6%) participants were currently pregnant, 15 (44%) participants had a livebirth in the prior three years, and 5 (15%) participants had a personal or partner miscarriage in the past three years.

**Table 2:** Participant characteristics among adult participants with a history of STI, in a sero-different relationship, and with recent or intentions for a personal or partner pregnancy.

Participant characteristic (n = 37)	Total		Women		Men	
	N	(Percent)	N	(Percent)	N	(Percent)
	Median	[Range]	Median	[Range]	Median	[Range]
Participant type						
Index participant	28	(76%)	18	(49%)	10	(27%)
Partner participant	9	(24%)	5	(14%)	4	(14%)
Median age	33	[22–50]	29	[22–40]	41	[27–44]
Living with HIV						
Yes	16	(43%)	3	(13%)	13	(93%)
No	20	(54%)	19	(83%)	1	(7%)
I don’t know	1	(3%)	1	(4%)	0	(0%)
Primary partner living with HIV*						
Yes	13	(46%)	10	(56%)	1	(10%)
No	11	(39%)	4	(22%)	9	(90%)
I don’t know	4	(14%)	4	(22%)	0	(0%)
Prior STI in lifetime						
Yes	32	(86%)	19	(83%)	13	(93%)
No	2	(5%)	2	(9%)	0	(0%)
I don’t know	3	(8%)	2	(9%)	1	(7%)
Number of sexual partners in the past year	1	[1–80]	1	[1–80]	1	[1–3]
Personal or partner pregnancy**						
No pregnancy	12	(34%)	7	(35%)	5	(36%)
Current pregnancy	2	(6%)	1	(5%)	1	(7%)
Livebirth in past 3 yrs	15	(44%)	8	(40%)	7	(50%)
Miscarriage in past 3 yrs	5	(15%)	4	(20%)	1	(7%)

*Among 28 index participants

**Among 34 participants

### Overview of qualitative findings

This analysis was guided by the Health Literacy Skills Framework [19]. The HLS Framework outlines that specific factors affect one’s health literacy skills, which then go on to affect health-related behaviors and outcomes. Participants reported that their “sources of STI information” were primarily from the general community (friends, family members, and acquaintances) and medical professionals. Participants also believed that “STI transmission” could occur through sexual as well as non-sexual mechanisms. Additionally, participants had different perceptions of each STI pathogen. For example, syphilis was specifically believed to be passed down genetically from parent to child, while gonorrhea carried significant stigma. Finally, participants reported uncertainty regarding “STIs and pregnancy”, specifically whether STIs affected pregnancy outcomes and whether antenatal STI treatment was safe.

### Domaine 1: Sources of STI information

#### Theme 1: Sources of STI information

Participants pieced together information from different sources to better understand STI transmission, prevention, and treatment. The general community was a commonly cited source of STI information and often the first resource, followed by medical professionals and participation in research. A minority of participants described learning about STIs through media sources such as the radio and through school.

*“I can first have a discussion with the community people*. *In business areas*, *such conversations are common*. *During such conversations*, *I get to know people’s thinking about STIs and their knowledge too*. *Then after I can visit some doctors that I know in private and government hospitals for more questions that I may have*.”

Female, age 35

*“Sometimes we would go [participate in research studies] because of the tea*, *sweets*, *and gifts that we would be given*, *which made me want to learn more in the end*. *I felt that I liked it…The chances to learn may be many*, *and I get to learn too because I move a lot*.”

Female, age 37

*“Concerning STIs*, *even if you turn on the radio*, *they are always talking about gonorrhea and syphilis*…*And when it’s taught*, *it makes you fear it as it was taught about*.”

Male, age 43

### Domaine 2: STI transmission

#### Theme 1: STI transmission thought to occur through sexual and non-sexual practices

All participants identified sexual activity as a mode of sexual transmission with many citing “adultery” and the concept of having multiple partners as a risk factor. Abstinence, monogomy, and condom use were all noted to be STI prevention strategies. In addition to sexual transmission, participants reported non-sexual STI transmission modes further outlined below. Participants were generally confused by these two STI transmission modalities with some participants believing that only certain STIs were transmitted non-sexually and others believing that all STIs could be spread through sexual and non-sexual modes.

*“Sometimes STIs confuse me because they can be acquired not necessarily through having sex*. *So*, *there is no way I can tell you that this kind of infection is through having sex*. *This is through my experience*. *I get them not because I had sex*, *but it just happens and I find out that I have them*. *Also*, *the times I have gone for checkups*, *the doctors have not told me that it is a sickness related to adultery*. *So*, *there is no way I can explain it*.”

Female, age 35

#### Theme 2: STI transmission thought to be through sharing items and space with people with STIs including clothing and bathrooms

Participants described being in close proximity and sharing house-hold items and clothing as a mode of STI transmission. Dirty clothes and wash basins are examples of items that were thought to transmit STIs to sexual and non-sexual partners including schoolmates, children, and strangers. Additionally, sharing bathrooms and the splash-back of urine and dirt from public toilets was considered to be a common mode of STI transmission with many participants recommending use of a personal bucket for urination as a way to prevent STI acquisition and transmission. The risk of acquiring STIs from urinating in shared toilets was applied more generally to women than men. Participants reported being counseled on these non-sexual transmission modes by healthcare workers in clinic in the midst of their STI diagnosis.

*“The sharing of clothes especially these pants*, *where one will wear them and after*, *another will do so as well*, *including basins and sponges*. *Yeah*, *those are the ways I think a person can get an STI*.”

Female, age 27

*“I don’t know about syphilis much*. *But concerning women*, *when they are urinating in the toilets they say that she can get the infection from there*, *so she has to be careful and know that she urinates in a clean place*. *As men*, *we are lucky that we urinate while standing*, *but if a woman is urinating down here she can get different sicknesses such as syphilis and candida*.”

Male, age 34

#### Theme 3: STI transmission associated with being dirty

Similarly, the idea of dirt and a lack of personal hygiene, devoid of sexual activity, as risk factors for STI acquisition were also common.

*“Being around sweaty people and rubbing shoulders so when the sweat falls on you*, *you can be infected with the STI*. *Or if you sleep with a very sweaty man*, *you can get the STI*.”

Female, age 27

*“Even if he has not committed adultery outside*, *sometimes the sickness can come from the dirtiness in men*, *like syphilis and candida*, *because in most cases these sicknesses are brought about by men that don’t care to clean themselves*. *The way he has been at work is the way he will go to bed*, *and sometimes he’ll come home while drunk and he’ll not have the strength to take a bath*. *Like the conditions of drunkards*, *he wants to come and have sex with you*, *so tomorrow you will wake up with the itchiness*.”

Female, age 24

### Domaine 3: STI pathogens

#### Theme 1: Syphilis is thought to be transmitted both sexually and genetically

Participants described confusion over how syphilis is transmitted. While many participants understood that syphilis could be transmitted through sexual activity, most participants also described inheriting syphilis “genetically” from their parents and grandparents. Participants were unsure whether sexually transmitted versus “genetic” or “inborn” syphilis were one or two separate infections. The concept of “inborn” syphilis also prompted some participants to question whether this type of syphilis could be cured and the subsequent value of treatment.

*“Well the way I know syphilis*, *I know it as a sickness that is inborn or genetic*. *Either your grandfather had syphilis or your mother that gave birth to you*, *so you find that it passes through blood genetically*, *and you find that it disturbs you a lot*. *In most cases*, *you find that syphilis is caused by adultery too*, *so now it confuses me because if it’s through blood then how does it come to be sexually transmitted*?”

Female, age 37

*“Let’s say I have a specific sickness and then I tell it to my friend who is also sick*. *So*, *you find that she has syphilis*, *but there is nothing she can do about it because she was born with it*, *so she is comforted by that*. *So even if she treats it*, *she will not get cured*.”

Female, age 25

#### Theme 2: Candida believed to be an STI

Most participants named Candida as an STI, though some voiced uncertainty over the nuances of this infection, including whether Candida could affect men, women, or both. Participants also noted the non-sexual methods of acquiring Candida.

*“Hmm the STIs that I know*, *of course there is gonorrhea*, *HIV*, *we have syphilis*, *candida*, *and others*.”

Male, age 35

*“That it’s the women that normally get infected with it but I don’t know how it is transmitted…I don’t know if a man can be infected with it*, *can he*? *I don’t know about it*. *That candida can stop a woman from giving birth*."

Male, age 34

*“Now with candida*, *it is different from syphilis…And as we were taught about wearing non-dry nickers*, *for the germs that are in the nickers will easily infect you*, *or you find that some people share nickers like the school children*. *So with candida*, *it passes through that so you treat it but you still get it somewhere*.”

Female, age 30

#### Theme 3: Gonorrhea is highly stigmatized

Compared to the other curable STIs, gonorrhea was generally thought to be transmitted through sexual contact only and therefore associated with increased stigma. One participant also notably reported being told that an active gonorrhea infection would protect against HIV acquisition.

*“At least the STIs like candida or syphilis*, *you will know that you have got it from urinating but with gonorrhea*, *you will know that you have got it from adultery*. *[Male partner] will have committed adultery because there is no other way he would have got gonorrhea…It was also hard for us because I was feeling sorry and hurt for a fellow wife*.”

Female, age 24

*“Well before*, *I don’t even know if they were trying to fool us but we would be told that when you have gonorrhea*, *you should leave it and stay with it because when you have it*, *you cannot have HIV/AIDS*.”

Male, age 43

### Domaine 4: STIs and pregnancy

#### Theme 1: Uncertainty whether STI medications are safe during pregnancy

While the majority of participants knew that STIs can be vertically transmitted to neonates, a few men were unaware of the deleterious effect of STIs on pregnancy. Additionally, when discussing neonatal STI infection, participants only referenced syphilis specifically, and it is unclear whether they knew of the risk of other neonatal infections. Notably, some participants were unclear whether STI treatment was safe to take during pregnancy and voiced concern over the medication effect on the fetus.

*“If I have been taught it by the doctor*, *and he tells me that it [STI treatment] has no effect on my pregnancy*. *I have no problem with it because I want to be cured*.”

Female, age 37

*“You find that when you go for treatment [during pregnancy]*, *you are not given enough medication*. *You are given the normal medication of a person who is not pregnant which she was not meant to use so you find that it affects the child inside so a miscarriage has to happen*.”

Female, age 30

*“I told them that my wife is pregnant and they asked me how many months of pregnancy she has*. *But she was 7 months pregnant so they said no and that they can’t put her on injections because they are strong*. *So*, *I went and told her and she got the injections later*. *She gave birth from here and after that*, *she was put on the dose and we had sex after she was done with her dose*.”

Male, age 34

## Discussion

This study explored STI knowledge and perceptions among men and women in sero-different HIV relationships, with recent or planned pregnancy, and with a prior self-reported STI diagnosis. Though participants learned about STIs from both the community and medical professionals, participants generally had several inaccurate ideas about STI transmission modalities and what pathogens constituted STIs. While sexual activity was a recognized method for STI transmission, participants incorrectly believed that sharing items, such as clothing, sharing spaces, such as toilets, and being dirty were also STI transmission modes. Further, participants mistakenly believed syphilis was transmitted genetically from parent to child and commonly thought Candida was an STI. Though the majority of participants realized STI treatment during pregnancy is appropriate, a few participants voiced concern over the side effects of STI treatment on the fetus and described delaying treatment of the mother until the post-natal period. Our data demonstrate widespread STI misconceptions highlighting opportunities for educational and counseling interventions.

Participants primarily reported learning about STIs from a combination of the general community including friends and acquaintances, medical professionals, and the media. Few studies have assessed sources of STI information among adults in resource-limited settings. Some studies examining university students’ STI information sources across settings from Ethiopia to Malaysia found that participants preferred the internet as their primary STI knowledge source [21, 22]. These findings contrast with our study where no participants reported the internet as a source of STI knowledge. This discrepancy is likely due to poor smartphone and computer access in our setting as well as our participants’ slightly older age [23]. A cross-sectional, mixed-methods study of adolescent girls and young women in the Nakivale refugee settlement in southwestern Uganda found that teachers and school were the most important source of STI knowledge [24]. These data indicate a potential for internet-based as well as school-based STI education interventions ideally focused on adolescents and young adults prior to or around sexual debut. School-aged children and adolescents in resource-limited settings often lack access to general sexual and reproductive health education with knowledge gaps that can persist into adulthood as exemplified among our participants [25]. A foundation of STI knowledge instilled in youth could then be supplemented by continuing education and counseling opportunities throughout adulthood in community, religious, and health centers when available.

In addition to sexual transmission, participants also described non-sexual STI transmission modalities including sharing items such as clothing, using public toilets, and being dirty. STIs inclusive of HIV have long been associated with the idea of dirt and being dirty [26–29] resulting in STIs requiring additional levels of counsel and care compared to other disease states. This idea of being “dirty” is sometimes used by healthcare workers to explain the disease state as “dirty blood”, especially when patients are asymptomatic [29, 30]. A qualitative study analyzing web-based transcripts from the American Sexual Health Association website found that clinicians focused on clinical and logistical STI information, while neglecting psychosocial STI information (information related to the psychological and social well-being of the individual) [31]. Similarly, a qualitative study of women with STIs and sexual health clinicians primarily from New Zealand found that participants benefited when clinicians acknowledged STI stigma and when they placed a reassuring value judgment on STI acquisition, as opposed to taking a neutral, non-judgmental stance [32].

While certain STI pathogens can persist for several hours on damp materials, such as towels and toilet seats, it is almost impossible for such fomites to transmit genital STIs to adults [33]. More commonly, these fomites spread to more susceptible sites, such as gonococcus causing conjunctivitis [34, 35] and more susceptible hosts, such as children, who due to physiologic factors, are potentially susceptible to STI acquisition through non-sexual exposures such as dirty clothing, communal baths, and caregiver hands [36]. These nuances of non-sexual STI transmission have understandably led to confusion among many people across the world. Studies in Uganda, Nigeria, and India have similarly found beliefs of non-sexual STI transmission [17, 26, 37]. Though most people know that STIs are spread through sexual contact, STI stigma likely compels people to favor non-sexual over sexual transmission modes [38]. Furthermore, numerous participants in our study reported that healthcare workers had counseled them that their STIs were likely acquired non-sexually. We do not know whether such counseling stems from a lack of knowledge or whether this counseling is purposeful in that it likely reduces STI-related stigma and facilitates partner notification [39]. Further research is needed to better understand and support healthcare workers’ STI knowledge and counseling strategies, especially in areas with limited resources.

Specific STI transmission and prevention beliefs directly affect the internal and external stigma associated with each STI as well as how participants think about treatment and cure. Participants in our study believed that syphilis could be both genetically inherited as well as sexually transmitted. This belief that syphilis can be genetically passed down is likely associated with the concept of vertical transmission. Without education and counseling on the difference between passing on disease states to offspring via vertical infection as opposed to genetic DNA, these two ideas could easily be conflated. Two other Ugandan studies have described the idea of syphilis as a genetic versus sexually-transmitted pathogen [17, 18], and it is unclear whether this concept extends to other areas of the world. Limited syphilis POC diagnostics adds to this confusion. In most RLS, only treponemal POC tests are available which often remain positive for a person’s lifetime. Thus, in many RLS, once a participant has a diagnosis of syphilis, they are unable to receive adequate tests that clarify treatment response, cure, or reinfection. Accurate, non-treponemal POC tests are needed to understand treatment response, cure, and reinfection. The combination of conflating vertical and genetic transmission with limited POC testing capacity likely led participants to voice uncertainty over the ability to achieve syphilis cure and the need for treatment. The idea of syphilis as a genetic disease also likely played a role in participants believing that syphilis cannot be cured and contributed to low syphilis stigma.

Many participants in our study incorrectly believed vaginal candidiasis to be an STI. Inclusion of vaginal candidiasis as an etiology of the WHO-classified vaginal discharge syndrome may be contributory [40]. Additionally, our participants attributed non-sexual risk factors leading to candida infection to STI transmission in general, further indicating the conflation of candida with other STIs. Finally, given that there is no Runyankole (local, predominant language) word for candida, participants in our setting may have confused the words candida and chlamydia. These misunderstandings may lead to candida having increased stigma and shame in our study setting compared to other study settings where candida is widely recognized as not being associated with sexual transmission [41].

Further, gonorrhea is recognized as primarily being transmitted via sex and therefore carries the greatest stigma of the three STI pathogens that participants were commonly able to name. Much research groups all STIs and reproductive tract infections together, but our findings demonstrate participant nuance in how they consider various pathogens. More research is needed to better understand how participants in various settings view individual STIs as there can be a wide range of perceptions. Additionally, greater education efforts are needed to teach patients accurate transmission modalities associated with various pathogens while simultaneously prioritizing stigma reduction efforts.

A small, but notable, number of participants did not realize the deleterious effect STIs have on pregnancy and that STI treatment during pregnancy is appropriate and safe, and these knowledge gaps were specifically striking among men. There is a dearth of research exploring these topics. Among 280 pregnant women surveyed in Gambia, 94% had poor STI knowledge in general [42]. Additionally, numerous studies across the world highlight the gaps in male reproductive health knowledge and subsequently recommend increasing male participation in perinatal and sexual and reproductive healthcare [43–47]. Of our participants who did not receive STI treatment during pregnancy, all reported being told by their healthcare provider that treatment was unsafe during pregnancy. Researchers in Brazil found that antenatal clinic providers had low competency in congenital syphilis knowledge [48]. The WHO has made the elimination of mother-to-child transmission of syphilis a top priority [10]. To better understand the gaps in antenatal STI care, a better understanding of provider knowledge and comfort with STI testing, treatment, and counseling is needed with improved implementation of trainings and refresher trainings [49, 50]. Additionally, participants in our study named only syphilis as causing neonatal morbidity and mortality and would benefit from further education on the neonatal transmission of other STIs.

### Limitations

Consistent with other qualitative studies, this study had several limitations including a small sample size and lack of generalizability. We focused our recruitment on participants living in rural, southwestern Uganda and thus from a specific cultural context. Additionally, participants were recruited from the MRRH Healthy Families Clinical Program that focuses on sero-different HIV couples counseling, thus our participants may have had increased HIV and STI knowledge compared to the general public. Our recruitment relied on participant self-report as well as clinical documentation of prior STI, so some participants may have had reproductive tract or genitourinary infections that that they confused with STIs (such as participants with candida as mentioned above). Finally, we were unable to track the percentage of participants that we approached versus those who participated in the study. Though this manuscript focuses on a specific population in a specific cultural setting, the lessons of centering specific populations’ STI beliefs to create tailored educational and counseling programs can be applied anywhere.

## Conclusion

Understanding STI knowledge and perceptions is key to understanding and treating the high STI prevalence and incidence across the world. Local cultural contexts and healthcare systems clearly influence all aspects of sexual and reproductive healthcare including STI care. The complicated nature of STIs has led to understandable confusion and assumptions in settings without formal sexual and reproductive healthcare education. In our study setting in southwestern, rural Uganda, we found that participants were unsure which pathogens constituted STIs and incorrectly believed STIs could be transmitted through numerous non-sexual methods. A minority of participants were also not aware that STIs during pregnancy could result in poor pregnancy outcomes nor were they aware that STI treatment during pregnancy was appropriate and safe. More robust counseling and education beginning ideally prior to sexual debut will allow men and women to understand the symptoms, transmission modalities, and appropriate treatments necessary for STI cure. Such education efforts would ideally take place both inside the healthcare system and also in the broader community (such as schools, religious organizations, social media), where much STI information is acquired. Additionally, future work is needed to better understand healthcare provider knowledge and counseling strategies to provide appropriate support to an often-overburdened healthcare system.

## Supporting information

S1 Text Inclusivity in global research. (DOCX)
